# The Development of SARS-CoV-2 Variants: The Gene Makes the Disease

**DOI:** 10.3390/jdb9040058

**Published:** 2021-12-15

**Authors:** Raquel Perez-Gomez

**Affiliations:** Translational Genomics Group, Institut Universitari de Biotecnologia y Biomedicina BIOTECMED, Universitat de Valencia, 46100 Valencia, Spain; perezgomezr@gmail.com

**Keywords:** SARS-CoV-2 genome, Spike protein, receptor binding domain (RBD), escape mutation, neutralizing antibodies (nAbs), variant of concern (VOC), COVID-19 vaccines

## Abstract

A novel coronavirus (SARS-CoV-2) emerged towards the end of 2019 that caused a severe respiratory disease in humans called COVID-19. It led to a pandemic with a high rate of morbidity and mortality that is ongoing and threatening humankind. Most of the mutations occurring in SARS-CoV-2 are synonymous or deleterious, but a few of them produce improved viral functions. The first known mutation associated with higher transmissibility, D614G, was detected in early 2020. Since then, the virus has evolved; new mutations have occurred, and many variants have been described. Depending on the genes affected and the location of the mutations, they could provide altered infectivity, transmissibility, or immune escape. To date, mutations that cause variations in the SARS-CoV-2 spike protein have been among the most studied because of the protein’s role in the initial virus–cell contact and because it is the most variable region in the virus genome. Some concerning mutations associated with an impact on viral fitness have been described in the Spike protein, such as D614G, N501Y, E484K, K417N/T, L452R, and P681R, among others. To understand the impact of the infectivity and antigenicity of the virus, the mutation landscape of SARS-CoV-2 has been under constant global scrutiny. The virus variants are defined according to their origin, their genetic profile (some characteristic mutations prevalent in the lineage), and the severity of the disease they produce, which determines the level of concern. If they increase fitness, new variants can outcompete others in the population. The Alpha variant was more transmissible than previous versions and quickly spread globally. The Beta and Gamma variants accumulated mutations that partially escape the immune defenses and affect the effectiveness of vaccines. Nowadays, the Delta variant, identified around March 2021, has spread and displaced the other variants, becoming the most concerning of all lineages that have emerged. The Delta variant has a particular genetic profile, bearing unique mutations, such as T478K in the spike protein and M203R in the nucleocapsid. This review summarizes the current knowledge of the different mutations that have appeared in SARS-CoV-2, mainly on the spike protein. It analyzes their impact on the protein function and, subsequently, on the level of concern of different variants and their importance in the ongoing pandemic.

## 1. Introduction

Human coronaviruses (HCoVs) are zoonotic pathogens that belong to the Coronaviridae family (order Nidovirales). They are characterized by envelopes that present projections that make them resemble a crown (in Latin ‘corona’) under electron microscope virions [1,2,3,4]. There are four coronavirus genera (α, β, γ, δ) within the Coronaviridae family. Seven species of human betacoronaviruses lead to diseases. Of them, HCoV-229E, HCoV-HKU1, HCoV-NL63, and HCoV-OC43 cause mild respiratory apparatus infection with efficient treatment [5]. The other three, MERS, SARS-CoV-1, and SARS-CoV-2, cause severe disease that can lead to fatal consequences [6,7]. Since the start of 2020, SARS-CoV-2 has spread around the globe, leading to a pandemic that has already caused more than four million deaths in less than two years.

All viruses mutate, including SARS-CoV-2. Adaptive mutations in its genome are altering its pathogenic potential, and these mutations can make therapy, vaccine development, and pandemic control difficult. This is a review of the different genetic variants of SARS-CoV-2 that have emerged to date around the globe and a summary of the known consequences of the virus fitness and the human disease. First, the virus’ structure, way of action, and genomic stability are introduced, which is essential to understand the meaning of the different mutations that have appeared in SARS-CoV-2. Secondly, how some mutations affect the development of the virus and how a particular set of mutations may provide improved viral functions are analyzed. Finally, we discuss the impact of different variants regarding the disease severity and if they could impact a vaccine’s efficacy or human development.

### 1.1. SARS-CoV-2 Structure

All CoVs have non-segmented genomes consisting of a positive-sense large single-stranded RNA (ssRNA) with a 5′ cap structure and a 3′ poly-A tail. The SARS-CoV-2 genome encodes 26 proteins (Table 1). Approximately two-thirds of its genome consists of one large open reading frame (ORF1ab), translated into pp1a or pp1ab polyproteins. These polypeptides are processed by a virally encoded main protease (Mpro, also 3CLpro or nsp5) and a papain-like protease (PLpro or nsp3) into 16 non-structural proteins (nsp1–16). Most of them seem to be essential for virus replication and for the adaptation of the virus to a new host [2,8,9]. nsp12 is the RNA-dependent RNA polymerase (RdRP) that, along with many other nsps, constitutes a replicase–transcriptase complex. It remains unclear what every nsp’s function is, but some evidence has been collected from the study of other HCoVs [2,3].

**Figure 1:**
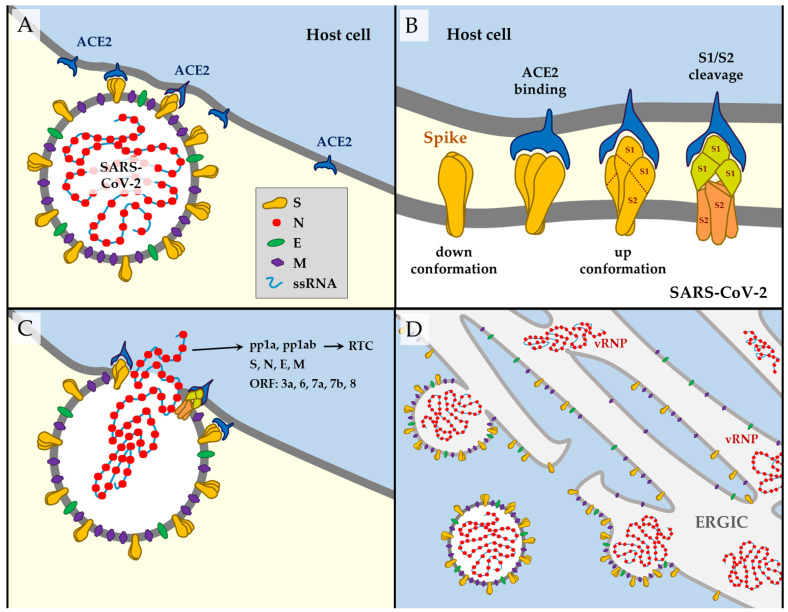
(**A**) SARS-CoV-2 structure of the SARS-CoV-2, comprising an ssRNA and 4 structural proteins interacts with the host cell through the ACE2 receptor (dark blue). Blue field represents inside the host cell, and yellow field outside the cell. (**B**) Changes in the spike during the virus-cell interaction. At the left, the inactive or ‘down’ conformation, reluctant to bind ACE2. Spike–ACE2 binding produces a conformational change in the S towards the ‘up’ configuration. This is followed by S1/S2 cleavage by host enzymes and activation of the entry to the cell. (**C**) Viral ssRNA enters the cell and produces pp1a and pp1ab, which will provide the RTC plus structural proteins (S, N, E, M) and accessory proteins (ORF). (**D**) N proteins are assembled with the new replicated viral ssRNA to form the vRNP. New viruses bud from the ERGIC and are released from the cell.

The remaining third of the genome comprises genes encoding the structural proteins [9]—spike (S), envelope (E), membrane (M) and nucleocapsid (N) (Figure 1A)—and six predicted accessory proteins—ORF3a, ORF6, ORF7a, ORF7b, ORF8, and ORF10 [10]. According to experimental data, there is no evidence of ORF10 expression, so this protein is unlikely to be expressed. The other ORFs could have essential and diverse roles, given the analysis of other coronaviruses [2], but their functions remain largely unexplored (Table 1). The transcriptome of SARS-CoV-2 is poorly understood, but it seems clear that the most transcribed proteins are the structural ones. Besides the canonical transcripts, a wide variety of incomplete transcripts have been documented, as well as post-transcriptional modifications in the viral RNAm, including events of RNA fusion. However, the meaning of these elements remains unclear [10].

From a mutational point of view, nonstructural proteins have attracted less attention than the structural components because the proteins on the virus surface represent the preferential targets of the host immune response. The S glycoprotein is the most exposed, located on the outer surface of the virion. It determines the initial interaction with the cell and most likely represents the primary determinant of host and tissue tropism. It is divided into S1 and S2 subunits (Figure 1B). S1 subunit is further subdivided into a receptor-binding domain (RBD) and an N-terminal domain (NTD) [21].

Given that adaptive mutations could be naturally selected in broader populations, studying SARS-CoV-2 genomic variants and their tracking with time might help us understand viral evolution, behavior, and development.

### 1.2. SARS-CoV-2 Way of Action

**Figure 2:**
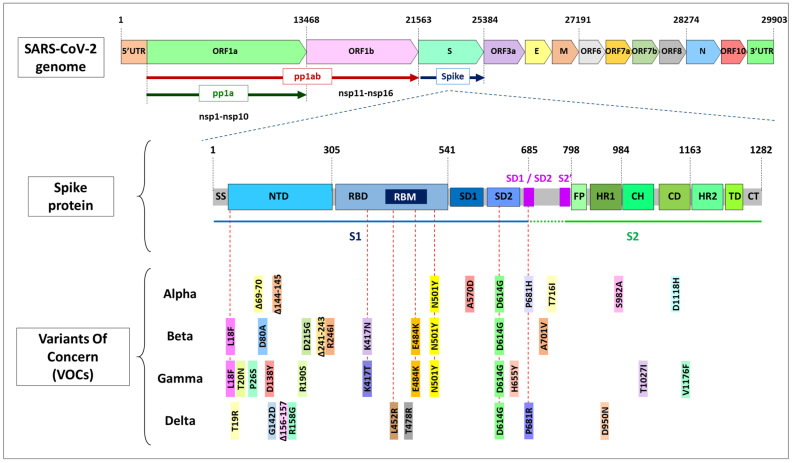
On the **top**, a schematic view of the SARS-CoV-2 genome that spans almost 30 kb. Polyprotein pp1a produces nsp1–nsp10 and pp1ab generates nsp1–nsp16. In the **middle**, the detailed structure of the spike protein. SS: signal sequence, NTD: N-terminal-domain, RBD: receptor binding domain, RBM: receptor binding motif, SD: subdomain, FP: fusion peptide, HR: heptad repeat, CH: central helix, CD: connector domain, TD: transmembrane domain, CT: cytoplasmic tail. Cleavage of the spike protein in SD1/SD2 yields spike subunits S1 and S2, activating the virus entry in the host cell. At the **bottom**, a schematic representation of the mutations included in the VOCs until September 2021. Red dotted lines point out mutations of concern that are shared by different variants.

The SARS-CoV-2 initiates its viral cycle with attachment to the host cell—mediated by the spike, the main factor responsible for the infection (Figure 1A). S trimers recognize the angiotensin-converting enzyme 2 (ACE2) receptor to perform the initial interaction with the host cells [22,23,24,25,26]. This occurs mainly on the respiratory epithelium, such as type II alveolar epithelial cells, where this receptor is abundant [27,28,29]. There are three different conformational states of the homotrimeric S glycoprotein (Figure 1B). The inactive or ‘down’ configuration corresponds to the receptor-inaccessible state. Upon binding to ACE2, it adopts a protruding ‘up’ conformation that promotes several rounds of cleavage by furin and other cell proteases in the S1/S2 site (see also Figure 2). This converts the S protein into an amino (N)-terminal S1 subunit and a carboxyl (C)-terminal S2 subunit responsible for virus–cell membrane fusion [3,30,31]. A second cleavage site, S2′, is highly conserved among coronaviruses and its cleavage is essential for successful infection [32,33,34,35]. The spike undergoes significant conformational changes towards an open state that facilitates attachment to the host cell [36]. The release of S1 triggers a structural rearrangement to fuse the viral membrane with the host cell membrane (Figure 1C) [33]. Both the plasma membrane (direct entry) [37] and endosomal [38] viral fusion pathways have been reported for SARS-CoV-2 entry into cells.

After penetration of the viral RNA into the host cell’s cytoplasm, pp1a and pp1ab are synthesized to form a replication–transcription complex (RTC) pertaining to the biosynthesis of new ssRNA molecules and viral proteins [2]. N supports replicating the viral genome in the cytoplasm and encloses novel viral RNA to form viral ribonucleoprotein complexes (vRNPs) [39]. The N protein wraps around the RNA genome, encapsulated within an envelope associated with the S, E and M proteins (Figure 1D) [40]. During the maturation process steps, cytoplasmic vRNPs are assembled with S, E, and M proteins within the endoplasmic reticulum–Golgi intermediate compartment (ERGIC). Mature virions bud at the ERGIC membrane, forming vesicles that are subsequently released from the host cell via exocytosis [8,9,39]. Structural proteins E and M facilitate viral transport and the assembly, budding, and release of SARS-CoV-2 virions from infected host cells [11,41].

Respiratory transmission is the primary route of infection; thus, the respiratory system is the predominant target for SARS-CoV-2. Nevertheless, it can affect other major organ systems, which could explain the multisystemic failure with fatal outcomes observed in some patients [16]. Environmental factors, such as temperature, population, and air pollution, affect viral spreading and mortality [42]. A few studies suggest a correlation between the extent of ACE2 expression in individuals and the clinical outcome of SARS-CoV-2 infection, especially in elderly populations and those with comorbidities [17,18,43].

### 1.3. Mutations in Coronavirus ssRNA

Mutations in the virus’s genome occur naturally due to mistakes in replication. Mutation rate, understood as the frequency of single nucleotide change per genome per viral cycle, ranges from 10^−8^ to 10^−6^ for DNA viruses and from 10^−6^ to 10^−4^ for RNA viruses [44]. The mutations are called synonymous when there is no change to the amino acid encoded by the gene and non-synonymous when the protein acquires an amino acidic change due to the mutation. They are mostly inconsequential and, in the case that they do change a protein, they tend to harm the virus more than improve it. Only a few of them can enhance the virus’ functions and its ability to spread or cause disease by affecting cell tropism or pathogenicity [45]. An extra advantage to the virus in terms of infectivity, transmissibility (see Box 1), or resistance against treatments or the immune system [46,47] will allow it to spread faster throughout the population.

Box 1 **Glossary of terms related to epidemic and viral evolution.**

**Viral fitness:** refers to reproductive success, understood as the number of copies that a virus produces of itself. In other words, it is the capacity of a virus to produce infectious progeny in a given environment.

**Viral load:** is the concentration of viral particles in a particular tissue or fluid. The faster and more efficient is the viral replication, the higher the viral load is expected to be.

**Infectivity:** is the capacity of viruses to enter the host cell and exploit its resources to replicate and produce infectious viral particles, which may lead to infection and subsequent disease in the human host.

**Transmissibility:** is the capacity to pass from one host to another; in other words, the power to spread. It is expected to increase if the viral load increases.

**Contagion:** is the communication of disease from one person or organism to another.

**Severity** (of a disease): it refers to the seriousness of the symptoms produced by the contagion of a disease.

Generally, the nucleotide substitution rates of RNA viruses are high [45,48]. Because too many mutations could be deleterious, RNA genomes are usually small, less than 20 kb [18]. Despite this, CoVs, and Nidovirales generally, have exceptionally long genomes (up to 32 kb) thanks to the presence of a high-fidelity system for replication [16,17,18,49,50]. It consists of a set of RNA-processing enzymes with exoribonuclease (ExoN) and, possibly, endoribonuclease (NendoU) activities. nsp14 encoded by the *ORF1b* is an enzyme possessing two activities: RNA 3′-to-5′ exoribonuclease [51] and N7-guanine methyltransferase (N7-MTase) [52]. The interaction of nsp10/nsp14 increases ExoN activity 35-fold [11,12], providing an efficient proofreading function during the replication of RNA strands. The N7-MTase performs the 5′ methylation of the viral GpppA cap, providing efficient RNA translation. Such a methylation process has been described for nsp14 [15] and the nsp10/nsp16 complex [13,14,53], and it would change the RNA cap of the virus to mimic host mRNAs and prevent the recognition of viral RNAs by host defenses. Altogether, it is clear that the SARS-CoV-2 level of mutation is relatively low. However, it is also clear that human coronaviruses are undergoing antigenic evolution in response to immune pressure [54].

Viruses obtain the genetic variability necessary to improve a virus function in two ways. First, the antigenic drift consists of the successive accumulation of mutations until there is a noteworthy change in the properties of any protein. Second, the antigenic shift occurs when different nucleotide strands recombine during virus replication, leading to a new combination of mutations [55]. This phenomenon is characteristic of the influenza virus, and it represents a continuous challenge for population immunity. Fortunately, there is no evidence of an antigenic shift in SARS-CoV-2, and coronaviruses in general are not prone to it. However, it has recently been documented that there could be recombination events during the replication of viral RNA mediated by nsp14 [56]. Although the mutations produced are expected to be either neutral or deleterious, a small number of them are positively selected, as they confer a fitness advantage [57]. Vast numbers of infected worldwide allow these advantageous mutations to arise recurrently in the population. Moreover, long convalescent patients have shown to be an ideal environment for the virus to propitiate the accumulation of mutations in a short time [58]. Adaptive mutations in the viral genome can alter the virus’s pathogenic potential. Even a single amino acid exchange can drastically affect a virus’s ability to cause an increase in infectivity, transmissibility (see Box 1), or provide the capacity to evade the immune system, at least partially [2,59]. Mutations that can resist immune attacks to some extent are cataloged as escape mutations. They need to be identified and monitored, as they can render the virus resistant to neutralization by host antibodies originating both naturally by infection or artificially through vaccination.

As a response to evolutionary pressure, SARS-CoV-2 accumulates mutations at the rate of around two changes per month [60], often consisting of single-letter substitutions. This is shaped by natural selection that quickly picks up mutations [45,48]. This accumulation is directly favored in the context of vast amounts of people infected worldwide, providing enough possibilities for successful evolution [61] and giving rise to new lineages and virus variants.

## 2. Mutations in the *spike* Gene

The evolution rate of the *spike* is three times higher than the evolution rate across the entire SARS-CoV-2 genome but still within the range of other betacoronaviruses. The mutation rate is high enough to mutate on average every amino acid in the spike at least once in one patient [62]. The S protein, and particularly the RBD, has a central role in engaging the angiotensin-converting enzyme 2 (ACE2) receptor to mediate cellular entry [63] and is a potential target for neutralizing antibodies (nAbs) elicited by either vaccination or natural infection [27,64,65,66,67]. There is also an addition of O-linked glycans that flank the cleavage site and are unique to SARS-CoV-2 [6]. Only a few RBD amino acids seem critical for binding to ACE2 receptors, determining the host range of SARS-CoV-like viruses [68].

The S protein possesses two surface areas of high mutagenic plasticity: the receptor-binding domain (RBD), where 17 residues make contact with the human ACE2, and the supersite in its N-terminal domain (NTD) [69]. Spike mutations can potentially facilitate better affinity or binding and improve the entry efficiency into the host cell. Increased infectiousness is commonly related to higher viral load (see Box 1) in patients and, subsequently, increased transmissibility [70]. Moreover, the spikes are exposed to the virus surface, making them a key site targeted by human antibody immunity [54,71,72,73,74]. Overall, there is a substantial selection pressure over this protein that could explain why the spike RBD is the most variable part of the SARS-CoV-2 genome [8,11] and why some of its variations are considered to be of concern [75].

The analysis of mutated versions of the RBD domain shows that, despite the face that most of the mutations do not affect spike properties, a few of them are considered of concern and can improve the virus functions [76,77]. The positions at which amino acid substitutions are present at the highest frequency are close to the RBD–ACE2 interface.

Although it is important to address which mutations may affect the severity of COVID-19, the impact of such an effect is challenging to predict; it remains difficult to assess precisely to what extent the mutations may confer disease severity (see Box 1), especially given the multiple factors that contribute to patient prognoses, such as age and underlying medical conditions.

### 2.1. D614G

The genetic evolution of SARS-CoV-2 is unclear before the pandemic. Starting in 2020, soon after the emergence of the zoonosis, the D614G mutation, where amino acid D (aspartic acid) was replaced by G (glycine), appeared to be associated with higher transmissibility [27]. D614G is located in an area where S1/S2 successive cleavages occur that are necessary for the entrance of the virus into the cell (Figure 1B). While the wild-type S trimer opens only one RBD on average, the G614 trimer opens two or all three RBDs [78]. The analysis of the S protein structure using both cryo-electron microscopy [79] and computational modeling analysis [80] found that bearing D614G favors an ‘open’ configuration that facilitates ACE2 binding [11] and increases the spike density in the virion surface [81]. As a consequence, the viral infectivity is enhanced [23,47,82]. Experiments performed using pseudoviruses pointed out that the presence of this mutation makes cell infection up to ten times more efficient in a human lung cell line and airway tissues, also being at greater levels in the upper airways of infected hamsters [79,83,84]. Furthermore, the D614G mutation reduces furin cleavage, thereby lowering the risk of premature S1 shedding, and it enhances the thermal stability of the spike.

Despite the slight change in the viral sequence, the fitness advantages for the virus are profound. While inter-person transmission becomes more likely, neither disease progression nor neutralization by anti-spike antibodies are significantly affected by the D614G mutation [85,86]. The estimated increase in transmission offers the virus a selective advantage that makes it globally dominant [87]. The D614G mutation is the hallmark of all variants (Table 2) and delimitates the founding of the B1 lineage [88]. It has been prevalent during the whole pandemic and, at present, almost all new infections of COVID-19 contain this mutation, which is present in all variants of concern (VOCs).

### 2.2. N501Y

This mutation corresponds to an amino acid located in the RBD, near the tip of the spike, where it seems to change the protein’s shape to be a tighter fit with human cells. The residue 501 is at the RBD–ACE2 interface, and the N501Y change results in increased affinity of the S protein for the ACE2 receptors, enhancing the viral attachment and the subsequent entry into the host cells [71,88]. Consequently, this mutation contributes to the virus’s improved infectivity, and it has been associated with faster transmission and possible adverse illness in young and healthy individuals [133]. In fact, N501Y has been shown experimentally to result in one of the highest increases in ACE2 affinity conferred by a single RBD mutation [76]. Still, it is not an escape mutation [134].

The improved binding affinity of spike for the ACE2 receptor is one of the defining factors that explain the high cell infectivity of SARS-CoV-2 and the fast expansion of this N501Y in the population [95], a mutation that has appeared recurrently in many different strains and it is present in some of the most relevant variants [135].

### 2.3. E484K

This amino acid substitution, E instead of K in position 484, is located close to the tip of the coronavirus spike and produces a change in the RBD area that alters the protein’s shape. Even though the S1 movements favor the RBD-up conformation in the E484K mutant [36], this mutation has shown neutral to very mild effects on RBD–ACE2 binding.

Nevertheless, the E484K substitution alone has been shown to confer resistance to neutralization by several nAbs [74,86,136,137,138,139,140], and it is associated with immune evasion where neutralization by some plasma is considerably reduced [71,72,94]. In fact, there is much evidence supporting the fact that the E484K mutation enables the virus to escape some people’s immune responses [141], sometimes being impervious to convalescent’s serum [59,71,73,74] and escaping even a potent polyclonal serum targeting multiple neutralizing epitopes [74,76,122,142]. As happened with N501Y, mutation E484K has emerged recurrently in many different lineages, such as Beta and Gamma, pointing out that this mutation is favored by evolution [135]. This adaptive advantage has allowed virus strains bearing it to spread quickly through human populations. The importance of this position is further underscored by the convergent appearance of the E484Q mutation in the Indian B.1.617 lineage (Table 2).

### 2.4. Other RBD Mutations

A series of other mutations have been identified in the RBD [6] that provide resistance to nAbs and plasma from convalescent or vaccinated individuals. The substitution L452R can impair neutralization by several nAbs and convalescent plasma [29,74,122,123] and emerged independently in different lineages, such as the Delta and Epsilon variants [143]. The amino acid L452 does not directly contact ACE2 but lies just beside Y453, which is involved in receptor binding [69,131]. Mutation Y453F, along with N439K, G446V, K444E, and S477N, among others, which are located at the interface between the S1 and ACE2, have been shown to partially interfere with antibody binding and neutralization [29,71,76,122,142,144,145,146]. N439K has also been shown to enhance the binding affinity for the ACE2 receptor [76,146]. Close to them within the RBD, K417N, and K417T mutations have been repeatedly described to protect against binding to certain monoclonal antibodies [55]. Nevertheless, both K417N and K417T are expected to moderately decrease ACE2-binding affinity [76,147,148]. The main impact of the K417N mutation seems to be its ability to destabilize the RBD-down conformation (Figure 1B), thereby increasing the propensity of the open configuration [36]. Several studies point out that the combination of K417N + E484K + N501Y may cause a more significant decrease in neutralization than any single mutation by itself [36,129,149,150].

### 2.5. P681 Residue

Different mutations have been observed in this residue, such as the P681H mutation in the Alpha variant, P681R in Delta and ΔP681 in the Indian lineage B.1.617 (Table 2). The P681 site is located near the S1/S2 furin cleavage point. Its processing guarantees fusion with the membrane posterior to the spike–ACE2 interaction, thus allowing the virus entry into the cell [151]. It has been shown that artificial deletions in the S1/S2 site produce attenuated virus variants [152]. In fact, an insertion in position 681–684 can alter the viral function [153], suggesting that P681 may be under intense selective pressure.

### 2.6. NTD Deletions

RBD is immunodominant, although there is evidence for a substantial role of NTD in antigenicity [154]. NTD mutations converge allosterically on regions that enable the Spike to escape some nAbs. Deletions in the NTD have been observed repeatedly in the evolution of SARS-CoV-2, and they have been shown to change NTD antigenicity [73,107,155]. Some recurrently deleted regions within the NTD have been identified: Δ69–70, Δ141–144, Δ144–145, Δ146, Δ210 and Δ243–244 associated with a certain capacity to escape antibody neutralization [108,155,156,157]. The former is also related to the failure of the three S-target RT-PCR assay [158] and, subsequently, to the difficulty of SARS-CoV-2 detection. Its appearance is recurrent and often co-occurs with N439K, Y453F, and N501Y mutations [97], suggesting a selective advantage and possible epistasis between mutations, which should be further examined.

Unlike substitutions, deletions cannot be corrected by proofreading activity, which may accelerate adaptive evolution in SARS-CoV-2.

### 2.7. Mutations out of the spike Gene

Additional profound changes outside the *spike* gene started to be reported [159,160]. To date, there is a long list of mutations identified in SARS-CoV-2 by sequencing, including substitutions, deletions, and insertions, summarized in databases like CoV-GLUE (http://cov-glue-viz.cvr.gla.ac.uk/, accessed on 15 November 2021) where they can be consulted. Unfortunately, very little is known of the biological meaning of most mutations found. An analysis of GISAID sequences has identified a strain with a nine-nucleotide deletion in the *nsp1* gene that might affect the C-terminal region of the protein involved in the regulation of viral replication [161,162]. Nsp1, also known as the leader protein (Table 1), is central to inhibiting the antiviral innate immune response, particularly the expression of interferon-alpha. Extensive deletion in the *ORF7a* gene [163] and a deletion in the *nsp2* gene [164] have been detected clustered in European populations, but their impact is unknown.

## 3. SARS-CoV-2 Lineages. Classification of Variants: VOC and VOI

More than five million genome sequences have been deposited in open-source platforms such as GISAID (https://www.gisaid.org/, accessed on 2 December 2021), Nexstrain (https://nextstrain.org/, accessed on 2 December 2021) [135] and NCBI Virus (https://www.ncbi.nlm.nih.gov/labs/virus/, accessed on 2 December 2021). Their phylogenetic analysis highlights multiple clusters of related genomes, defined as clades, based on a set of common mutations. Lineages are analyzed, organized [165] and made available in public sites such as Pango (https://cov-lineages.org, accessed on 15 September 2021). Clade O was the ancestral type described in Wuhan [8,23]. Starting in 2020, it diversified into a more prevalent clade 19A (clade L) and clade 19B (clade S) [166]. A new clade bearing mutation D614G, called A2a or Clade G, identified in February 2020, became the founder of the B1 lineage and spread globally [77,167].

The variants of SARS-CoV-2 are defined by a particular genetic profile and a certain origin (Table 2). The Centers for Disease Control and Prevention (CDC) [90], the ECDC [91], and the World Health Organization [89] have independently established a classification system for distinguishing them into variants of concern (VOCs) and variants of interest (VOIs). The variants of interest (VOIs) are defined as those ‘bearing specific genetic markers that could be related to enhanced transmissibility or virulence, a reduction in neutralization by antibodies obtained through natural infection or vaccination, the ability to evade detection, or a decrease in the effectiveness of therapeutics or vaccination [27]. VOCs have already proven to fulfil these criteria and, because they disperse rapidly through populations, they are considered a threat to public health. Since September 2021, due to the fast expansion of the Delta variant, most of the other variants have been displaced and are now considered unimportant for the institutions mentioned. Most of these variants are now classified as variants under monitoring (VUM) for the WHO, variants being monitored for the CDC or even de-escalated for the ECDC (Table 2).

The variants accumulate a series of mutations that characterize them and, surprisingly, some of the VOCs share mutations that repeatedly appear in different virus strains and locations [135]. The recurrent occurrence of the same mutations and their fast spread into other populations suggests the existence of selection advantages for them. It points out a phenomenon of convergent evolution [91,107,157]. The variability accumulates better in the context of chronic infections or in previously immunized individuals [58,155,168,169,170], which could benefit the spontaneous co-occurrence of the same mutations in different lineages.

Despite the virus’s sluggish mutation rate, researchers have catalogued more than 12,000 mutations in SARS-CoV-2 genomes. It has been estimated that two SARS-CoV-2 viruses collected from anywhere in the world differed by an average of 10 changes [58], primarily single substitutions, along with small deletions. Unfortunately, scientists can spot mutations in RNA sequences faster than they can make sense of their meaning and their implications in pathogenesis.

As expected, variants with improved efficiency in replication, transmission or infection spread very fast all over population. The variants have been assigned with different nomenclatures. They were initially defined by the date of first appearance and their level of concern (i.e., VOC-202012/01), or by any of the mutations they bear (i.e., 20I/501Y.V1) or according to their genetic Pango lineage (i.e., B.1.1.7). In June 2021, the World Health Organization introduced a new naming system [171] based on Greek letters (Table 2).

Since the onset of the SARS-CoV-2 pandemic, few VOCs have been considered—only Alpha, Beta, Gamma, and Delta, which are associated with enhanced transmissibility and increased virulence [16]. Although Delta has dispersed worldwide and is now the focus of attention, all variants require special care and surveillance [172].

### 3.1. Variants of Concern (VOCs)

A variant of concern (VOC) is a mutated strain that possesses higher transmissibility, causes more severe disease progression, increases mortality, escapes antibody neutralization, and/or evades detection. Alpha, Beta and Gamma are no longer considered VOCs, but they were considered so, and it is important to analyze the factors that made them spread through populations.

#### 3.1.1. Alpha (B.1.1.7 Lineage)

In September 2020, a new variant called Alpha was described as powerfully associated with fast-growing outbreaks across the UK, particularly London and Kent [94], promoting a four-fold increase in cases in just ten weeks. This lineage (clade GR) is considered 43–90% more transmissible than the Wuhan strain [3,95,97,98] and has mutated at a much faster rate than other variants according to the ECDC [173]. The increased Alpha variant fitness made it prevalent worldwide in only a few months [148], outcompeting different previous strains and becoming particularly abundant in Europe and North America (https://cov-lineages.org, accessed on 20 October 2021).

This variant bears a few mutations that potentially affect viral function (Figure 2) [98]: D614G, N501Y, P681H and ∆69–70, among others. As explained above, mutation N501Y lies in a critical contact residue in the RBD that enhances virus binding to human ACE2 [76,95]. Mechanistically, the amino acid 501 is involved in establishing the hydrophobic interactions with the ACE2 receptor, and Y501 provides a stronger interaction [96]. This could destabilize the RBD-down conformation, thereby adding more open RBDs to the D614G effect [36]. It is predicted that RBD altered conformation by N501Y explains the 5 to 10-fold higher affinity compared to N501 versions. Its location and the recurrent appearance of N501Y in different virus strains suggests that it is a major determinant for the increased Alpha variant transmission [3].

In addition, mutation P681H, which is immediately adjacent to the furin S1/S2 cleavage site in spike [63,174], could facilitate the processing of the spike protein, and thus improve binding to ACE2. Consequently, it could make the entry to the cell faster and more efficient. Although P681H initially raised much interest, it has not yet been found to significantly impact viral fitness [175] (see Box 1).

Exposed in the NTD loop of the Alpha variant, the previously described ∆69–70 mutation has been linked to a certain level of immune escape in immunocompromised patients and can enhance viral infectivity and transmissibility [97,155]. The observed reductions in the neutralization activities of the NTD-directed nAbs [3,98,102,103] are also attributable to the Δ144–145 mutation (Figure 2).

Altogether, the Alpha variant showed an enhanced spike-ACE2 affinity, better access to the cell, and a certain but low resistance to some nAbs. This leads to an increase in the replication, which pushes the Alpha variant to a higher viral load observed in the upper airways both in a hamster model and in human epithelial cells [133]. This improved infectivity could explain the observed augmented fitness for the Alpha variant [176,177].

Early reports found no evidence to suggest that this variant has any impact on the severity of disease or vaccine efficacy [71,72]. Nevertheless, the higher viral load observed in patients infected with the Alpha variant could be ultimately related to the increased infectivity and transmissibility, therefore explaining the rise in the percentage of fatal cases [94,95,99]. A large matched cohort study performed in the UK, among others, reported an increased mortality hazard ratio of patients infected with Alpha [99,100,101].

Despite being able to escape a small subset of nAbs, the Alpha variant has shown minimal impact on susceptibility to monoclonal antibody treatments and neutralization by convalescent and post-vaccination sera [178]. However, the detection in February 2021 of E484K acquisition in some Alpha sub-lineages raises concerns about this variant’s capacity to overcome the attack of antibodies [102].

#### 3.1.2. Beta (B.1.351 Lineage)

Towards the end of 2020, rising concern was reported in South Africa regarding lineage B.1.351 [104,105], which bears some worrying mutations in the *spike* gene—D614G, N501Y, E484K, and K417N (Figure 2)—with proven functional significance [129,179,180]. These mutations might explain the dominant spread of this variant in the region, becoming of significance not only in South Africa but also in North America and several European countries (https://cov-lineages.org, accessed on 20 October 2021).

Like the Alpha variant, the Beta variant presents an enhanced system for the entry to the cell provided mostly by D614G and N501Y. The increase has been mainly attributed to the N501Y mutation in both variants [36]. The Beta RBD has been shown to bind ACE2 with 4.62-times greater affinity than the wild-type [106]. However, the neutralization activities of some antibodies directed against the Beta Spike were seriously impaired or even wholly abolished [109], which is attributed to the presence of the E484K mutation and, to a lesser extent, to the K417N substitution [142], both in the RBD. Moreover, an allosteric effect of the NTD mutations (L18F, D80A, D215G, Δ243–244 and R246) in the spike interaction with ACE2 has been suggested, because the binding of the Beta spike to the NTD-directed nAbs was dramatically reduced [103,107,108,109,110,111]. In fact, the combination K417N + E484K + N501Y, considered the most efficient in terms of immune scape [36,129,149,150] with the addition of R246I, makes this variant extremely dangerous.

Overall, these mutations generate significant conformational changes that avoid the binding of a different set of antibodies. This worries health experts because clinical trials of vaccines show that they offer less protection against this variant than against other ones [115]. According to the ECDC, people who recover from different variants may not be able to fend off the Beta variant because their antibodies will not be efficient enough against its spike [112,113,114].

#### 3.1.3. Gamma (P.1 Lineage or B.1.1.28.1)

In December 2020, a variant circulating in Manaus, Brazil, was identified in international travelers. This coincided with a resurgence of infections and a rapid increase in the number of COVID-19 hospitalizations despite the high seroprevalence: 76% of the population had previously been shown to have antibodies against the virus [115,116]. Variants containing multiple advantageous mutations are solid contenders for enabling reinfections and lowering vaccine efficacy globally, as in Manaus. This Gamma variant became abundant in all of America and Europe (https://cov-lineages.org, accessed on 23 October 2021).

Like many other B.1 lineages, the Gamma variant also has the B.1-defining mutation, D614G, related to increased spike on the virus surface [79]. This variant shares with the Beta variant some mutations with functional significance, which were acquired independently: E484K, N501Y, and K417T (instead of K417N found in Beta) located in the RBD, and L18F in the NTD. In fact, it has been proposed that K417N could provide a slightly improved ACE2 binding compared to K417T [3,36,179]. These mutations are responsible for the improved infectivity and the capacity for immune evasion observed in the Gamma variant, which dramatically contributed to its rapid spread through the American continent [109,113,117,118,119].

Despite sharing all these mutations, the Gamma variant seems less resistant against nAbs than Beta. The reason for this is not yet apparent. Still, it has been suggested that the great variability in the NTD between these variants, with allosteric influences from other mutations (T20N, P26S, D138Y, R190S; Table 2), could contribute to the observed differences. The most exposed amino acids are more susceptible to being the target of nAbs.

#### 3.1.4. Delta (B.1.617.2 Lineage)

The ancestral lineage B.1.617 is not a variant but a cluster of sequences within clade G that share the common signature mutations: G142D, L452R, E484Q, D614G, and P681R [77]. It is divided into three sublineages [129,168]: B.1.617.1 (Kappa variant), B.1.617.2 (Delta variant) and B.1.617.3 (Table 2). The E484Q substitution, which may be functionally similar to E484K [136], is present in the B.1.617.1 (Kappa) and B.1.617.3 sublineages and the ancestral B.1.617 but is likely to have reverted and is absent in the Delta lineage [90,93].

The main particularity of the Delta variant is the increased fitness. This variant replicates more efficiently, thus increasing its infectivity and transmissibility. In fact, individuals infected with Delta have viral loads up to 1260 times higher [125] compared to other strains. Instead of E484Q, Delta bears T478K, which falls within the same epitope region that targets potent neutralizing monoclonal antibodies [102]. This mutation is unique to this lineage, and it could be the determinant of Delta’s success, but it remains unknown whether it improves the virus fitness.

L452R, defined as an escape mutation [29,74,122,123], has been shown to promote a much higher viral replication in non-human cell cultures [181], which would correspond with the better fitness of the Delta variant observed in populations. However, this mutation has not shown a relevant impact in other variants, such as Epsilon [143].

P681R is located in the S1/S2 furin-cleavage site [182], the same residue affected in the P681H substitution found in the Alpha variant. It has recently been documented that P681R provides a pre-active state that could facilitate virus–cell binding and, in general, cell infection [124]. It has been proposed that viruses bearing P681R fuse with the plasma membranes of uninfected cells, a critical step in disease, almost three times faster than control ones. Recent studies demonstrate that the Delta virus behaves more efficiently during cell entry, promoting cell fusion and augmented syncytium formation [183]. The observation that the Delta spike can cause more cell-to-cell fusion may suggest that the virus causes more tissue damage, thus being more pathogenic than previous variants. In fact, the viral spread via syncytium formation could contribute to the efficient inter-and intra-host spread of this variant. This would explain why in cultured human–airway epithelial cells infected with equivalent amounts of Delta and Alpha viral particles, Delta rapidly outcompetes the Alpha variant, mimicking epidemiological patterns played out globally in human populations. Nevertheless, researchers suggest that there must be more mutations to make the difference for Delta, because other variants carrying P681R, such as the Kappa variant, do not share this high efficiency during infection. The different results across studies are not conclusive, and there are still many aspects to clarify about this mutation [168].

The genetic profile of the Delta variant is considerably particular, as it does not bear the typical mutations observed in other VOCs, except D614G (Figure 2). It has been suggested that the Delta variant presents a noticeably different receptor-binding interface compared to the other variants [184]. Nonetheless, the positions of mutations in the S protein showed a similar overall distribution in the Delta variant to other VOCs (Figure 2). The D950N mutation is mapped to the S trimer interface [93], suggesting that this mutation may contribute to the regulation of S protein dynamics [183]. Clustered in the NTD domain, the Δ156–157, Δ157–158 [77], and G158R mutations, unique to the Delta variant, map to the same surface as the Δ144–145 and Δ241–243 deletions in the Alpha and Beta variants, respectively (Figure 2). These altered residues are targeted by most anti-NTD nAbs [117].

The mutations in Delta strains reported may differ depending on the resources consulted [77,93,185]. The description of mutations that occurred in the *ORF1ab* locus is especially unclear for the Delta variant, and often they do not match from one source to another (Table 2). Although mutations of the *spike* gene have not been analyzed in detail, they could have consequences in terms of replication efficiency and contribute to explaining the substantial viral load found in individuals infected with this variant.

In clinical terms, it has been observed that in individuals infected with the Delta variant, the virus becomes detectable four days after exposure, compared with an average of six–seven days among people infected with other variants. This observation, along with the increased viral load, suggests that Delta replicates much faster, which could explain why superspreading events are even more common and likely to affect more people [126]. Indeed, the short incubation period makes contact tracing even more difficult [127,128]. The increased capacity to be amplified in patients and transmitted between people makes this variant very good at spreading and, in fact, it is already outcompeting all the other variants in most countries. The Delta variant was initially considered a variant of interest. However, this variant rapidly spread worldwide, prompting the WHO to classify it as a VOC in May 2021.

A unique combination of mutations characterizes the Delta genetic profile. Despite being different from previously successful variants, it has managed to disperse worldwide, becoming the only Variant of Concern since September 2021. While it has been described that the combination of K417N + E484K + N501Y is the most resistant to nAbs [36,129,149,150], the Delta variant, lacking all three of them, has displaced all the other VOCs, becoming prevalent in most countries towards the mid of 2021 [186]. It remains unclear whether the presence of T478K or P681R could be decisive, causing this variant’s increased strength. Another possibility suggested to explain the higher viral load found in tissues infected with the Delta variant would depend on variations within the N protein. This protein binds to the viral ssRNA after replication to form the vRNP (Figure 1D), and it has been suggested that the mutation N:R203M (Table 2) is involved in an improved assembly mechanism that would contribute to explaining the fast spread of the Delta variant [187]. Nevertheless, this fact would not explain why the variant Kappa and lineage B.1.617.3, which also harbor this mutation, did not have the same success as Delta.

### 3.2. Variants of Interest (VOIs)

The VOIs present specific genetic markers associated with improved virus functions, but they are not considered a threat to public health. The evidence about their impact is preliminary or subject to major uncertainty, and they are not dispersed significatively. In any case, they need to be monitored in populations because, based on their genetic profile, they could become a VOC under certain circumstances. A summary of the most relevant VOIs is shown in Table 2.

Among these is the Epsilon variant, which comprises two lineages (B.1.427/B.1.429) and was identified in California in January 2021. It is characterized by the L452R mutation, which was expected to give an advantage when spreading over other variants, being more contagious than earlier forms [27,105,130]. Some studies claimed that this variant hda a modest capacity for immune escape [131]. Nevertheless, Epsilon was deescalated from a VOC in June 2021 due to the evidence about its impact being unclear, a significant decrease in the variant circulation, and vaccines had proven to be effective against it [89,90,91].

The variant Eta corresponds to lineage B.1.525, first appearing towards the end of 2020 in Nigeria. It carries E484K and Δ69–70 mutations. In some strains, it bears Q677H, whereas in others it is Q677P. The Q677 location on the side of the S protein suggests that it might contribute to an easier entry and infection of human cells, but no clear evidence supports this claim. The variant Zeta, or lineage P.2, which independently acquired the E484K mutation, has been detected in many locations in Brazil, including in Manaus [132]. Unlike P.1 (Gamma), P.2 was removed from the VOI list due to declining prevalence and very few detections.

There are tens of other variants and lineages that have been detected in many different countries, many under surveillance, and others with no special interest. That is the case, for example, of variants Iota (lineage B.1.526), identified in New York in late December 2020 [188] and Theta (lineage P.3), both bearing mutations E484K and D614G, among others [89,90,91,112,171]. Because of the mutations they carry, these variants have the potential to reduce the neutralization by antibody treatments and vaccine sera. Still, they are not having a significant impact on virus fitness. The possibility of acquiring new mutations in these lineages cannot be discarded, so keeping the new infections under genetic surveillance is vital.

The Kappa and Delta variants and the lineage B.1.617.3 emerged in India from the same ancestor (B.1.617) [93], but neither Kappa nor B.1.617.3 have shown a clear impact on human health to be considered VOC. The Mu variant, classified as a VOI on 30 August 2021, has a constellation of mutations that indicate potential properties for immune escape. These mutations suggest a reduction in neutralization capacity of convalescent and vaccinee sera similar to that seen for the Beta variant, but this needs to be confirmed by further studies.

This was the situation until September 2021 when, surpassed by the expansion of the Delta variant, many other variants have been de-escalated (Table 2). The CDC [90] is not listing any VOI, but all variants are now considered variants being monitored (VBM), except the only one considered VOC: the Delta variant, which now globally prevails over all others. Alpha was de-escalated by the ECDC [91], besides many others, but Beta, Gamma, and Delta are still considered VOCs. For the WHO [89], Alpha to Delta variants are considered VOCs, but they only mention two VOI: Lambda and Mu. They include the rest of the lineages as variants under monitoring (VUM). This is a rapidly changing scenario and a clear example of how fast the genetics of the virus adapt to new situations, both improving their viral cycle functions and evading the immune response.

## 4. Escape Mutations and Vaccine Efficacy

While antiviral medication development has not been very successful, about one year after the pandemic’s breakout, there are at least 13 vaccines against SARS-CoV-2 in use [189,190]. All of them have been developed to train the immune system to recognize the S protein, which is immunodominant [191]. Two mRNA-based vaccines were developed by Pfizer and BioNTech (BNT162b2) and by Moderna (mRNA-1273). Oxford University developed the AstraZeneca vaccine (ChAdOx1 nCoV-19) based on a chimpanzee adenovirus-vector [192]. The Janssen vaccine (Ad26.COV2.S, by Johnson and Johnson), administered in a single shot, is based on an inactivated virus [193]. These are only a few of the vaccines that are now globally available for public use and they are likely the most widely scrutinized. They have proven to be safe, and they have already been administered to millions of people.

To date, the administration of vaccines has been shown to avoid fatal disease, but they cannot completely block the contagion. As herd immunity rises, whether, through infection or vaccination, a steady trickle of immune-evading mutations could help SARS-CoV-2 to establish itself permanently, potentially causing mostly mild symptoms when it infects individuals immunized from a previous infection or vaccination. Despite the successes in vaccine development, reports of mutations are increasing. Some of these mutations bypass the immunity provided by several vaccine candidates [86].

The efficacy of the BNT162b2 vaccine against the four VOCs has been proven. Neutralization of the Alpha and Gamma variants was roughly equivalent [194,195]. On the other side, the neutralization of Beta was vigorous but lower than the ancestral SARS-CoV-2 strain [196,197,198]. Chen and colleagues [199] reported that sera from BNT162b2-vaccinated individuals showed decreased neutralizing potency against Alpha (2-fold), E484K + N501Y + D614G recombinant (4-fold), and two chimeric SARS-CoV-2 strains encoding Beta (10-fold) and Gamma (2.2-fold) compared to the D614G original. These data fit with other data published for both mRNA vaccines tested [150,200], but they found no significant effect for K417N mutation alone. In addition, convalescent plasma obtained six months after SARS-CoV-2 infection was 0.5- to 20.2-fold less effective at neutralizing the K417N + E484K + N501Y combination [27,129,149,150]. In vitro analysis of serum samples obtained from individuals administered the mRNA-1273 vaccine shows no change in the neutralization of the Alpha variant. Conversely, the analysis showed a decrease in titers of nAbs against the Alpha + E484K variant, Beta, Gamma and Epsilon variants. The reduction in neutralizing titers was significantly lower in the Beta variant [120,121].

In the case of the AstraZeneca vaccine, a 9.5-fold reduction in nAbs has been shown against Beta compared to Alpha [109,180]. A two-dose regimen of the AstraZeneca vaccine did not confer enough protection against the Beta variant based on results from a multicenter, double-blind, randomized control trial [200]. Another trial showed that in vitro neutralization activity of this vaccine against the Alpha variant was red. The vaccine’s clinical efficacy was 70.4% compared to the 81.5% efficacy noted in previous variants [201].

The Delta variant is less sensitive to sera from naturally immunized individuals and partially, but notably, escapes neutralizing monoclonal antibodies and polyclonal antibodies elicited by previous infection with SARS-CoV-2 or by vaccination. A single dose of either the Pfizer or the AstraZeneca vaccines induced a barely detectable level (10%) of nAbs against the Delta variant. About 10% of the sera neutralized this variant. Nevertheless, a two-dose regimen generated high sero-neutralization levels against the Alpha, Beta and Delta. The two-dose effectiveness against the Delta variant was estimated to be around 60% for AstraZeneca [93,202]. Neutralization experiments indicate that antibodies elicited by the Pfizer and AstraZeneca vaccines are efficacious against the Delta variant, but about three to fivefold less potent than those against the Alpha variant [199,203,204,205]. Recent experiments have shown that the Moderna vaccine could be the most efficient and long-lasting of all vaccines, closely followed by the Pfizer formulation [206,207]. On the other side, the Janssen vaccine presents a significative reduced efficiency compared to mRNA-based vaccines, particularly in a single dose [208].

The emergence of resistant SARS-CoV-2 variants may nullify the effects of current COVID-19 vaccines. Nevertheless, COVID-19 vaccines can elicit not only nAbs but also SARS-CoV-2-specific CD4+ and CD8+ T-cell responses that are poorly characterized. Cellular immunity may be more cross-reactive than the humoral response. It has recently been reported that T-cell responses to the Alpha, Beta, Gamma and Epsilon variants did not differ from those to the ancestral strain of SARS-CoV-2 [209].

Unfortunately, in a scenario of concern due to the high levels of circulating virus, which is facilitating the appearance of new variants across the globe, it is impossible to discard the idea that the vaccine efficiency could diminish [59].

## 5. COVID-19 during Human Development

To date, limited evidence prevents us from attaining a clear picture of how SARS-CoV-2 infection by different genetic variants could differentially affect human development. It is known that the course in pediatric COVID-19 is milder than in adults, as children have a better prognosis and deaths are extremely rare. One of the most consistent and biologically plausible theories emerging in the literature regarding the mild (if any) disease SARS-CoV-2 in children has been associated with age-related differences in AC. However, this remains only a hypothesis [210]. On the other hand, aged people, patients with comorbidities, or pregnant women can suffer an especially severe COVID-19 that results in hospitalization [28,29,46]. In a multinational cohort study, it was observed that women with COVID-19 diagnosis were at an increased risk of a composite maternal morbidity and mortality index. Newborns of women with COVID-19 diagnosis had significantly higher severe neonatal morbidity index and severe perinatal morbidity and mortality index compared with newborns of women without COVID-19 diagnosis. This study indicates a consistent association between pregnant individuals with COVID-19 diagnosis and higher rates of adverse outcomes, including maternal mortality, preeclampsia, and preterm birth compared with pregnant individuals without COVID-19 diagnosis [211] More studies are needed to understand these phenomena.

In the particular case of the Delta variant, hospital systems in different countries report numbers of younger people, even babies, admitted to hospital and a growing prevalence of severe or critical illness [186,212,213]. This could be a consequence of the enormous viral load found in individuals infected with the Delta variant, and the improved capacities for cell entry worsening the disease symptoms, along with the fact that young people are not yet vaccinated in many populations. Physicians are urging pregnant women to vaccinate as the Delta variant surges, and many countries have already started vaccinating their youngest population [214]. Much more data are needed and interesting ongoing trials are covering the topic [215].

Although the most common symptoms are related to breathing problems, SARS-CoV-2 infections also affect the gastrointestinal tract, culminating in inflammation and diarrhea. The mechanisms related to these enteric manifestations are still not well understood, but it has been hypothesized that an mTOR-driven increased autophagy that leads to intestinal dysbiosis could explain these symptoms [216].

## 6. Conclusions

The context of a massive vaccination campaign and high incidence of infection is providing the perfect environment for the rise of new variants resistant to immune neutralization or bearing improved functions. The virus develops new tools in a fast evolutionary race in order to increase its fitness. Periodic genomic sequencing of viral samples will help to detect any new genetic variants of SARS-CoV-2 circulating in communities, especially in a global pandemic setting. Vaccines may need to be updated periodically to avoid a potential loss of clinical efficacy [151].

Currently, there is an increasing number of studies aiming to unveil the details of SARS-CoV-2 development; from the genes to the structure and from the structure to the function. Many studies are trying to understand how the disease progresses in tissues and how the infection could affect human development. New information, emerging daily, will shed some light on these mechanisms. New variants that will arise in populations must be surveilled.

## Figures and Tables

**Table 1:** SARS-CoV-2 genome structure. Polyproteins pp1a and pp1ab synthesize non-structural proteins nsp1–nsp16, which are responsible for the replication of ssRNA. The 3′ third of the genome contains genes that synthesize structural proteins and ORFs. Many of the resulting proteins still have unknown functions. Data were collected from NCBI’s public gene database (https://www.ncbi.nlm.nih.gov/gene accessed on 14 September 2021) and [2,10,11,12,13,14,15,16,17,18,19,20]. Adapted from [2].

Gene	Transcript	Protein Name(s)	Position in the Genome	Length (aa)	Function
5′UTR			1–265		
ORF1a	pp1ab, pp1a [10]	nsp1	266–805	180	Leader protein. Cellular mRNA degradation, inhibiting IFN signaling [2].
		nsp2	806–2719	638	Unknown.
		nsp3, PLpro	2720–8554	1945	Papain-like protease, adenosine diphosphate-ribose 1″-phosphatase. Blocks host innate immune response, promotes cytokine expression [2].
		nsp4	8555–10054	500	Double-membrane vesicles formation [2].
		nsp5, 3CLpro, Mpro	10055–10972	306	3-chymotrypsin-like Cys protease. Main protease. Mediates cleavages downstream of nsp4. Inhibits IFN signaling [2].
		nsp6	10973–11842	290	Restricting autophagosome expansion. Double-membrane vesicle formation [2].
		nsp7	11843–12091	83	Cofactor with nsp8 and nsp12 [2].
		nsp8	12092–12685	198	Replicase. Cofactor with nsp7 and nsp12. Primase [2].
		nsp9	12686–13024	113	Replicase. ssRNA-binding protein. Dimerization and RNA binding [2].
		nsp10	13025–13441	139	RNA synthesis protein. Scaffold and cooperation with nsp14 ExoN and nsp16 in methyltransferase activities [2,12,13,14,15].
ORF1ab	pp1ab [10]	nsp11	13442–13480	13	Endoribonuclease and 3′-to-5′ exonuclease [2].
		nsp12, RdRP	13442–16236	932	RNA-dependent RNA polymerase: replication and transcription of the viral genome. Primer dependent RdRp [2].
		nsp13	16237–18039	601	DNA and RNA helicase/NTPase, 2′-O-ribose methyltransferase. RNA 5′-triphosphatase. RNA helicase 5′ triphosphatase [2].
		nsp14	18040–19620	527	ExoN. 3′-to-5′ exonuclease. N7-guanine methyltransferase [2,11,12,15].
		nsp15	19621–20658	346	Endoribonuclease, 3′-to-5′ exonuclease. NendoU. Evasion of dsRNA sensors [2].
		nsp16	20659–21552	298	2′-O-ribose methyltransferase [16,17,18]. Avoids MDA5 recognition, negatively regulating innate immunity [2].
S		Spike (S)	21563–25384	1273	Structural protein; surface glycoprotein. Mediates virus–host cell binding.
ORF3a		ORF3a	25393–26220	275	Ion channel activity (viroporin) activates the NLRP3 inflammasome. May play a role in virus replication and pathogenesis.
E		Envelope (E)	26245–26472	75	Structural protein. Envelope protein. Facilitates assembly and release of the virus. It has ion channel activity required for pathogenesis.
M		Membrane (M)	26523–27191	222	Structural protein. Membrane glycoprotein. Located in the transmembrane domain; it is the most abundant structural protein.
ORF6		ORF6	27202–27387	61	Suppression of both primary interferon production and interferon signaling [19].
ORF7a		ORF7a	27394–27759	121	Type I transmembrane protein.
ORF7b		ORF7b	27756–27887	43	Localize to the Golgi compartment.
ORF8		ORF8	27894-28259	121	Interferes with host antiviral mechanisms [20].
N		Nucleocapsid (N)	28274–29533	419	Structural protein. Nucleocapsid phosphoprotein protects the viral RNA genome and is involved in packaging RNA into virus particles.
ORF10		ORF10	29558–29674	38	Unknown. No transcripts identified [10].
3′UTR			29675–29903		

**Table 2:** Summary of main variants. Name(s), lineage, type, and distinctive mutations of the different variants mapped against the Wuhan-Hu-1 reference sequence (MN908947). The concern status is defined by the WHO [89], the CDC [90], and the ECDC [91]; de-esc: de-escalated. Mutations in the *spike* gene are underlined. Ϯ: Mutations in the Delta variant are undefined, particularly in the ORF1 region, and they differ from one database to another. In **bold letters** are those mutations documented by all different sources [77,92,93].

WHO Name [89]	Other Names	Lineage (Pango)	First Documented	Status WHO (*CDC)	Status from September 2021	Mutations: Amino Acid Modifications in Comparison to the Ancestral Wuhan-Hu-1 Sequence (NC_045512)	Impact of Mutations on Virus Functions
Alpha	VOC 202012/01, 20I/501Y.V1 (British variant)	B.1.1.7	UK, Sept-2020 [94]	VOC	WHO: VOC ECDC: de-esc CDC: VBM	PL:T183I, PL:A890D, PL:I1412T, nsp6:Δ106-108, RdRP:P323L, S:Δ69-70, S:Δ144-145, S:N501Y, S:A570D, S:D614G, S:P681H, S:T716I, S:S982A, S:D1118H, ORF8:Q27*, ORF8: R52I, ORF8:Y73C, N:D3L, N:R203K, N:G204R, N:S235F [92]	Increased affinity of S protein for ACE2 receptor, provided mostly by N501Y [72,95,96], enhances viral attachment and entry into host cells, making it 43–90% more transmissible [3,95,97,98]. Increased viral load [99] and increased severity of disease and mortality compared to previous circulating forms of virus variants [94,95,99,100,101]. Modest reductions in the neutralization activities of the NTD-directed nAbs [3,98,102,103]. Does not affect vaccine efficacy [71,72].
Beta	20H/501Y.V2 (Southafrican variant)	B.1.351	South Africa, Oct-2020 [104,105].	VOC	WHO: VOC ECDC: VOC CDC: VBM	nsp2:T85I, PL:K837N, 3CL:K90R, nsp6:Δ106-108, RdRP:P323L, S:L18F, S:D80A, S:D215G, S:Δ241-243, S:R246I, S:K417N, S:E484K, S:N501Y, S:D614G, S:A701V, ORF3a:Q57H, ORF3a:S171L, E:P71L, N:T205I [92]	D614G, N501Y, E484K, and K417N increase the binding affinity for the ACE receptors [36,106], increased risk of transmission and reduced neutralization by monoclonal antibody therapy, convalescent sera, and post-vaccination sera [103,107,108,109,110,111]. Reduced efficiency of vaccines, particularly low with AstraZeneca [111,112,113,114].
Gamma	P.1, 20J/501Y.V3, (Brazilian variant)	P.1, B.1.1.28.1	Brazil. Dec-2020 [115,116]	VOC	WHO: VOC ECDC: VOC CDC: VBM	PL:S370L, PL:K977Q, nsp6:Δ106-108, RdRPP323L, nsp13:E341D, S:L18F, S:T20N, S:P26S, S:D138Y, S:R190S, S:K417T, S:E484K, S:N501Y, S:D614G, S:H655Y, S:T1027I, S:V1176F, (ORF3a:G174C), ORF3a:S253P, ORF8:E92K, N:P80R, N:R203K, N:G204R [92] G142D [77]	D614G, N501Y, E484K, and K417T increase the binding affinity for the ACE receptors [36,106]. Genetic profile similar to Beta. Reduced neutralization by monoclonal antibody therapies, convalescent sera, and post-vaccination sera [109,113,117,118,119,120,121].
Delta	VUI-21APR-02, (Indian variant)	B.1.617.2	India. Dec-2020 [77,93]	VOC	VOC	nsp2:P129L, PL:P822L, PL:H1274Y, nsp4:A446V, nsp6:L37F, nsp6:V149A, **RdRP:P323L**, **RdRPG671S**, **nsp13:P77L**, nsp15:H234Y, S:T19R, S:G142D, S:Δ156-157, (S:Δ157-223 [77]), S:R223G, S:L452R, S:T478K, S:D614G, S:P681R, S:D950N, **ORF3a:S26L**, **M:I82T**, ORF7a:Δ39-49, **ORF7a:V82A**, ORF7a:L116F, **ORF7a:T120I**, **ORF8:Δ119-120**, **N:D63G**, **N:R203M**, **N:D377Y**, N:R385K [93] + PL:A488S, PL:P1228L, PL:P1469S, nsp4:V167L, nsp4:T492I, nsp6:T77A, nsp14:A394V, S:Δ157-223, ORF7bT40I [92] Ϯ	Delta presents a unique mutation T478K. Along with L452R, it provides immune escape [29,74,122,123]. P681R substitution supplies a pre-active state that could facilitate cell infection [124]. Delta replicates much faster, people become contagious before the starting of the symptoms, viral loads up to 1260 times higher [125]. Superspreading events are more common [126] and contact tracing even more difficult [127,128]. It has rapidly overcome other variants. Reduced neutralization by post-vaccination sera, particularly with AstraZeneca vaccine [120,121].
Lineage B.1.617		Ancestral B.1.617	India [77,93]			nsp1:Δ17, PL:Δ880, PL:A994D, RdRP:P323L, nsp13:Δ206, nsp13:Δ430, nsp14:Δ21, nsp15:A255V, nsp16:H186R, S:L452R, S:E484Q, S:D614G, S:Δ681, S:Δ1072, ORF3a:S26L, ORF3a:Δ96, M:I82S, ORF8:P93S, ORF8:E106Q, orf8:Δ121, N:Δ204, N:Δ215 [92]	Presents E484Q and L452R in the spike protein. Potential properties of immune escape and increased infectivity.
Epsilon	CAL.20C/ L452R (Californian variant)	B.1.427, B.1.429	USA. Mar-2020	VUM	WHO: VUM ECDC: de-esc CDC: VBM	nsp2:T85I, RdRP:P323L, nsp13:D260Y, S:S13I, S:W152C, S:L452R, S:D614G, ORFF3a:Q57H, N:205I (Exclusive of B.1.427: nsp4:S395T, nsp13:P53L) (Exclusive of B.1.429: nsp9:I65V) [92]	Expected to provide advantage at spreading [27,129,130] and modest capacity for immune escape [131]. Deescalated from VOC in June 2021, due to significant decrease in its circulation and vaccines have proven to be effective against it [89,90,91].
Zeta	VUI-202101/01	P.2, B.1.1.28.2	Brazil. Apr-2020 [132]	VOI*	WHO: de-esc ECDC: de-esc CDC: VBM	Spike mutations: L18F; T20N; P26S; F157L; E484K; D614G; S929I; and V1176F	Presents E484K in the spike protein. Potential properties of immune escape.
Eta		B.1.525	Nigeria and UK. Dec-2020	VOI	WHO: VUM ECDC: de-esc	PL:T1189I, nsp6:Δ106-108, RdRP:P323F, S:Q52R, S:A67V, S:Δ69-70, S:Δ144-145, S:E484K, S:D614G, S:Q677H, S:F888L, E:L21F, M:I82T, ORF6:Δ2-3, N:Δ3, N:A12G, N:T205I [92]	Presents E484K, Δ69–70, Δ144–145 in the spike protein. Potential properties of immune escape.
Theta	GR/1092K.V1	P.3	Philipines, Jan-2021 [Pango]		WHO: de-esc ECDC: de-esc	PL:D736G, PL:S1807F, nsp4:D217N, nsp4:L438P, nsp6:D112E, nsp7:L71F, RdRP:P323L, nsp13:L280F, nsp13:A358V, S:484K, S:N501Y, S:D614G, S:P681H, S:E1092K, S:H1101Y, S:1176F, ORF8:K2Q, N:R203K, N:G204R [92]	Presents E484K and N501Y in the spike protein. Potential properties of immune escape and increased infectivity.
Iota		B.1.526	New York. Nov-2020 [89]	VOI	WHO: VUM ECDC: de-esc CDC: VBM	nsp2:T85I, nsp4:L438P, nsp6:Δ106-108, RdRP:P323L, nsp13:Q88H, S:L5F, S:T95I, S:D253G, S:E484K, S:D614G, S:A701V, ORF3a:P42L, ORF3a:Q57H, ORF8:T11I [92]	Presents E484K in the spike protein. Potential properties of immune escape.
Kappa		B.1.617.1	India. Oct- 2020.	VOI	WHO: VUM ECDC: de-esc CDC: VBM	PL:T749I, nsp6:T77A, RdRP:P323L, nsp13:G206C, nsp13:M429I, nsp15:K259R, nsp15:S261A, S:E154K, S:L452R, S:E484Q, S:D614G, S:P681R, S:Q1071H, ORF3a:S26L, M:I82S, ORF7a:V82A, N:R203M, N:D377Y [93]	Presents E484Q, L452R, and P681R in the spike protein. Potential properties of immune escape and increased infectivity.
Lambda		C.37	Peru. Dec-2020.	VOI	WHO: VOI ECDC: VOI	nsp1:E102K, PL:A41V, PL:T428I, PL:D821N, PL1469S, nsp4:D217N, nsp4:D459N, 3CL:G15S, nsp6:L122S, nsp8:T148I, RdRP:P323L, nsp13:D105Y, S:S12F, S:Δ69-70, S:W152R, S:R346S, S:L452R, S:D614G, S:Q677H, S:A899S, M:I82T, ORF7b:A43S, N:R203K, N:G204R, N:G212V [92]	Presents L452R and Δ69–70 in the spike protein. Potential properties of immune escape.
Mu		B.1.621	Colombia. Jan-2021.	VOI	WHO: VOI ECDC: VOI CDC: VBM	PL:T237A, PL:T720I, nsp4:T492I, nsp6:Q160R, RdRP:P323L, nsp13:P419S, S:T95I, S:Y144S, S:Y145N, S:R346K, S:E484K, S:N501Y, S:D614G, S:P681H, S:D950N, ORF3a:Q57H, ORF3a:Δ256-257, ORF8:T11K, ORF8:P38S, ORF8:S67F, N:T205I [98]	Presents E484K and N501Y in the spike protein. Potential properties of immune escape and increased infectivity.
		B.1.617.3	India. Feb-2021 [93]	VUM, VOI*	ECDC: de-esc CDC: VBM	PL:A1T, PL:A1526V, PL:T1830I, 3CL:A194S, nsp6:A117V, RdRP:P323L, S:T19R, S:Δ156-157, S:R223G, S:L452R, S:484Q, S:D614G, S:P681R, S:D950N, ORF3a:Δ19-27+FS, ORF7a:V82A, N:P67S, N:R203M, N:D377Y [93]	Presents E484Q, L452R, and P681R in the spike protein. Potential properties of immune escape and increased infectivity.

## Data Availability

Not applicable.
